# GC-MS-based metabolite profiling of key differential metabolites between superior and inferior spikelets of rice during the grain filling stage

**DOI:** 10.1186/s12870-021-03219-8

**Published:** 2021-09-28

**Authors:** Xiumei Min, Hailong Xu, Fenglian Huang, Yidong Wei, Wenxiong Lin, Zhixing Zhang

**Affiliations:** 1 College of Life Science, Fujian Agricultural and Forestry University, 350002 Fuzhou, China; 2 Fujian Provincial Key Laboratory of Agroecological Processing and Safety Monitoring, Fujian Agriculture and Forestry University, Fuzhou, 350002 China; 3 Rice Research Institute, Fujian Academy of Agricultural Science, Fuzhou, 350018 China; 4 Key Laboratory of Crop Ecology and Molecular Physiology, Fujian Agriculture and Forestry University, 350002 Fuzhou, China

**Keywords:** Rice, Inferior spikelets, Grain filling, Metabolism, Trehalose

## Abstract

**Background:**

The asynchronous filling between superior spikelets (SS) and inferior spikelets (IS) in rice has become a research hotspot. The stagnant development and poor grain filling of IS limit yields and the formation of good quality rice. A large number of studies on this phenomenon have been carried out from the genome, transcriptome and proteome level, indicating that asynchronous filling of SS and IS filling is a complex, but orderly physiological and biochemical process involving changes of a large number of genes, protein expression and modification. However, the analysis of metabolomics differences between SS and IS is rarely reported currently.

**Results:**

This study utilized untargeted metabolomics and identified 162 metabolites in rice spikelets. Among them, 17 differential metabolites associated with unsynchronized grain filling between SS and IS, 27 metabolites were related to the stagnant development of IS and 35 metabolites related to the lower maximum grain-filling rate of IS compared with the SS. We found that soluble sugars were an important metabolite during grain filling for SS and IS. Absolute quantification was used to further analyze the dynamic changes of 4 types of soluble sugars (sucrose, fructose, glucose, and trehalose) between SS and IS. The results showed that sucrose and trehalose were closely associated with the dynamic characteristics of grain filling between SS and IS. The application of exogenous sugar showed that trehalose functioned as a key sugar signal during grain filling of IS. Trehalose regulated the expression of genes related to sucrose conversion and starch synthesis, thereby promoting the conversion of sucrose to starch. The difference in the spatiotemporal expression of *TPS-2* and *TPP-1* between SS and IS was an important reason that led to the asynchronous change in the trehalose content between SS and IS.

**Conclusions:**

The results from this study are helpful for understanding the difference in grain filling between SS and IS at the metabolite level. In addition, the present results can also provide a theoretical basis for the next step of using metabolites to regulate the filling of IS.

**Supplementary Information:**

The online version contains supplementary material available at 10.1186/s12870-021-03219-8.

## Background

Grain filling determines the yield and quality of cereal crops. Rice (*Oryza sativa* L.) is an important cereal crop that is critical for global food security [1]. However, grain filling in rice (especially the large-panicle varieties) exhibits differences in superior spikelets (SS) and inferior spikelets (IS), with the larger and heavier SS grains on the upper primary rachis branches and the smaller IS grains on the secondary rachis branches [2]. Poor IS grain filling limits the chance of achieving high yields in rice and severely impacts rice quality [3–5]. As a result, investigating the mechanism of poor grain filling and low grain weight in IS and establishing corresponding approaches to control and promote IS grain filling have become popular topics in crop science research.

Previous studies on the differences in grain filling between SS and IS at the genomic, transcriptomic, and proteomic levels have indicated that grain filling is a complex but orderly process and involves numerous changes in gene expression, protein expression, and protein modifications [6–9]. Metabolites are directly associated with traits; they can clearly reflect the quality of plant traits, including yield, nutritional components, and defense mechanisms, and play crucial roles in plant growth, development, and responses to biotic and abiotic stresses [10, 11]. Thus, to more comprehensively understand the mechanism that leads to the differences in grain filling between SS and IS, it is necessary to study their metabolites in depth and integrate this with genomics, proteomics, and phenotypic information for a systematic analysis. In recent years, metabolomics has been widely applied in studying the mechanism of rice grain development in response to the environment, for example, the differences in metabolites between *japonica* and *indicia* rice cultivars [12], the effect of high temperature on starch accumulation and amino acid metabolism [13], and the metabolic pathway of the formation of rice grain chalkiness [14]. Results from the studies mentioned above have provided new insights for understanding the mechanism of rice grain development (grain filling) at the metabolic level. However, to date no studies have analyzed the metabolites related to the differences in grain filling between SS and IS.

This study utilized gas chromatography-mass spectrometry (GC-MS), a non-targeted metabolomics approach, and identified 162 metabolites in rice grains. The differences in metabolites at different stages of grain filling between SS and IS were analyzed, and a key differential metabolite, trehalose, was identified. Functional verification showed that the change in the trehalose content was an important leading cause of the poor grain filling of IS. Results from this study could provide theoretical references for utilizing metabolites to control grain filling in IS in the field.

## Results

### Analyses of grain-filling dynamics and morphology of rice SS and IS

**Fig. 1:**
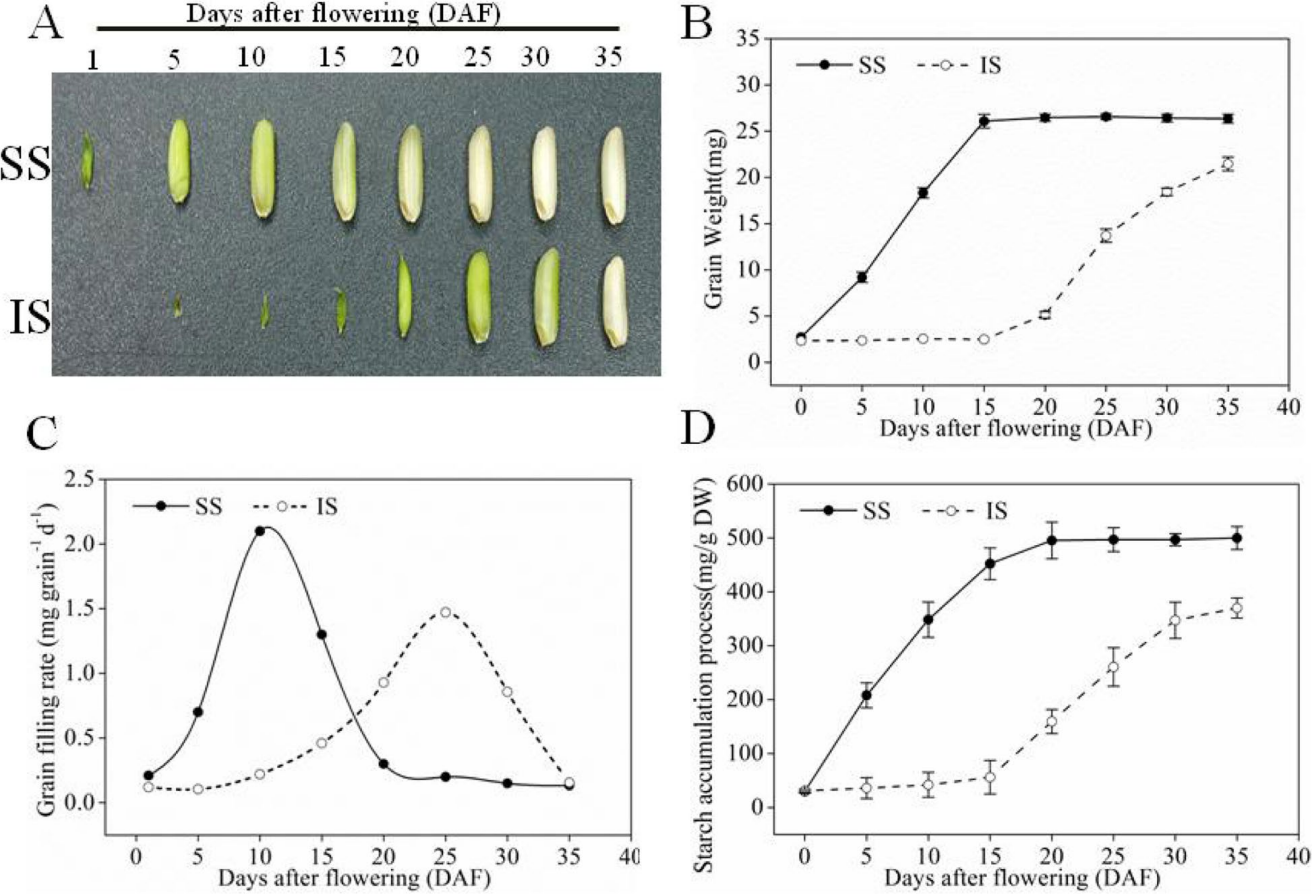
Grain-filling processes of superior spikelets (SS) and inferior spikelets (IS) of rice. **A** Developmental changes in representative SS and IS. **B** Grain weight of SS and IS. **C** Grain-filling rate of SS and IS. **D** Starch accumulation of SS and IS. The grain-filling rate was calculated according to Richards’ equation. Data in figures **B**, **C** and **D** are means and SD. of six independent measurements

In this study, the large-panicle rice cultivar ‘Jinhui No. 809’ was used. In its SS, grain filling and an increase in grain weight occurred rapidly from 0 to 15 days after flowering (DAF), while in the IS, the development was stagnant with no changes in grain weight or morphology from 5 to 15 DAF. After 15 DAF, grain filling was activated in IS, but at a slower grain-filling rate. The final 1000-grain weight of IS was also significantly lower than that of SS (Fig. 1A, B). Results from the Richards’ equation (Fig. 1C) showed that SS reached the maximum grain-filling rate at 10 DAF, while IS reached their maximum grain-filling rate approximately 25 DAF. Moreover, the maximum grain-filling rate of IS was significantly lower than that of SS. Starch accumulation process was consistent with the grain-filling process (Fig. 1D).

### Analysis of differential metabolites between SS and IS

**Fig. 2:**
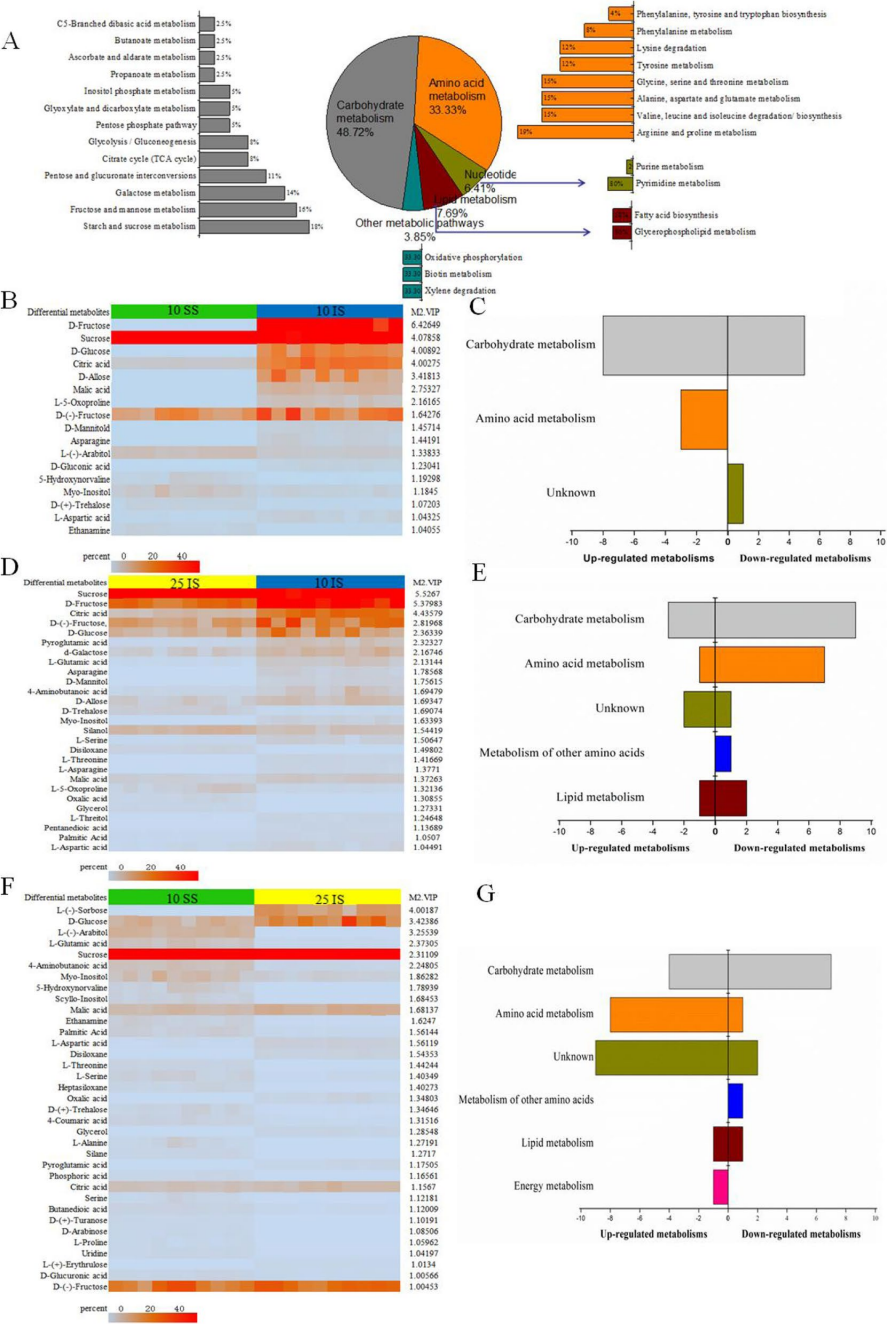
Analysis of differential metabolites between superior spikelets (SS) and inferior spikelets (IS). **A** Classification and distribution of the identified metabolites in SS and IS of rice. **B** Heat map presentation of the differences in metabolites between 10 DAF IS and 10 DAF SS. **C** Metabolic pathway distribution of differential metabolites between 10 DAF SS and 10 DAF IS. The left side represents the number of differential substances in the same pathway, where 10 DAF SS is higher than 10 DAF IS, and the right side is the opposite. **D** Heat map presentation of the differences in metabolites between 25 DAF IS and 10 DAF IS. **E** Metabolic pathway distribution of differential metabolites between 25 DAF IS and 10 DAF IS. The left side represents the number of differential substances in the same pathway, where 25 DAF IS is higher than 10 DAF IS, and the right side is the opposite. **F** Heat map presentation of the differences in metabolites between 10 DAF SS and 25 DAF IS. **G** Metabolic pathway distribution of differential metabolites between 10 DAF SS and 25 DAF IS. The left side represents the number of differential substances in the same pathway, where 10 DAF SS is higher than 25 DAF IS, and the right side is the opposite. The differential substances in Fig. **B**, **D**, **F** are arranged according to the VIP values from large to small. Each row represents a compound. The value of each compound represents the relative content directly normalized on the scale of the graph. Blue represents a low content, red represents a high content

In order to identified the differences in metabolites between SS and IS, 10 DAF SS (the active grain-filling period of SS), 10 DAF IS (developmental stagnancy phase of IS), and 25 DAF IS (the active grain-filling period of IS) were selected as study materials and GC-MS analyses were carried out. After removing interfering peaks and aligning peaks, data analysis of the metabolites from three sample groups showed that a total of 162 metabolites were detected, among which 78 had known metabolic functions and were involved in various metabolic processes (Fig. 2A). Thirty-eight metabolites were related to carbohydrate metabolism, comprising 48.72% of the total metabolites with known functions, such as starch and sucrose metabolism, the citric acid cycle, pentose and glucoronate interconversions, fructose and mannose metabolism, and glycolysis/gluconeogenesis. There were 26 metabolites related to amino acid metabolism, accounting for 33.3% of the total metabolites with known functions; they were mainly involved in secondary metabolic processes including glycine, serine, and threonine metabolism; valine, leucine, and isoleucine degradation; and aspartate and glutamate metabolism.

To screen for differential metabolites between SS and IS, the PCA model, OPLS model and S-plot (Fig. S1) among samples were constructed. Results showed that the data from all three sample groups were within the confidence interval, with good grouping results. Using the VIP value of the first principal component obtained from the OPLS model and *p*-value, differential metabolites were identified with VIP > 1 and *p* < 0.05. As a result, 17 differential metabolites were identified between SS and IS at 10 DAF (Table S1). As shown in Fig. 2B and C, most of these metabolites were soluble sugars and acid substances that were mainly associated with carbohydrate metabolism and amino acid metabolism. Among them, sugars such as sucrose, fructose, glucose, trehalose, and allose are involved in starch and sucrose metabolic pathways; hydroxyproline, asparagine, and aspartic acid are involved in amino acid metabolism. A comparison of the relative contents of these differential metabolites between SS and IS revealed that the majority of these sugars and acid substances exhibited higher contents in IS than in SS; only a few metabolites, such as sucrose and hydroxynovaline, had higher contents in SS.

Between the stagnant phase (10 DAF) and the active grain-filling stage (25 DAF) of IS, 27 differential metabolites were identified (Table S2, Fig. 2D, E). Among them, there were 13 metabolites involved in carbohydrate metabolism that were related to starch and sucrose metabolism, the TCA cycle, and fructose and mannose metabolism. There were 9 metabolites involved in amino acid metabolism, including glutamic acid, asparagine, and aminobutyric acid. A few metabolites were involved in lipid metabolism and nucleic acid metabolism. Furthermore, in IS at 25 DAF, compared to 10 DAF, the contents of most differential metabolites related to carbohydrate metabolism and amino acid metabolism decreased; only a few differential metabolites exhibited an increase in their contents, such as sucrose, trehalose, and silanol.

In total, 35 differential metabolites were identified between 10 DAF of SS and 25 DAF of IS (Table S3, Fig. 2F, G). The differential metabolites were mainly involved in the carbohydrate metabolism and amino acid metabolism. The results showed that the relative contents of soluble sugars, including sorbose, sucrose, fructose, glucose and trehalose in 25 DAF of IS were higher than in 10 DAF of SS. Comprehensive analysis of the differences in metabolites between SS and IS at the three grain-filling stages resulted in the identification of soluble sugars including sucrose, fructose, glucose, and trehalose.

### The dynamic changes in sucrose, fructose, glucose, and trehalose contents in SS and IS

**Fig. 3:**
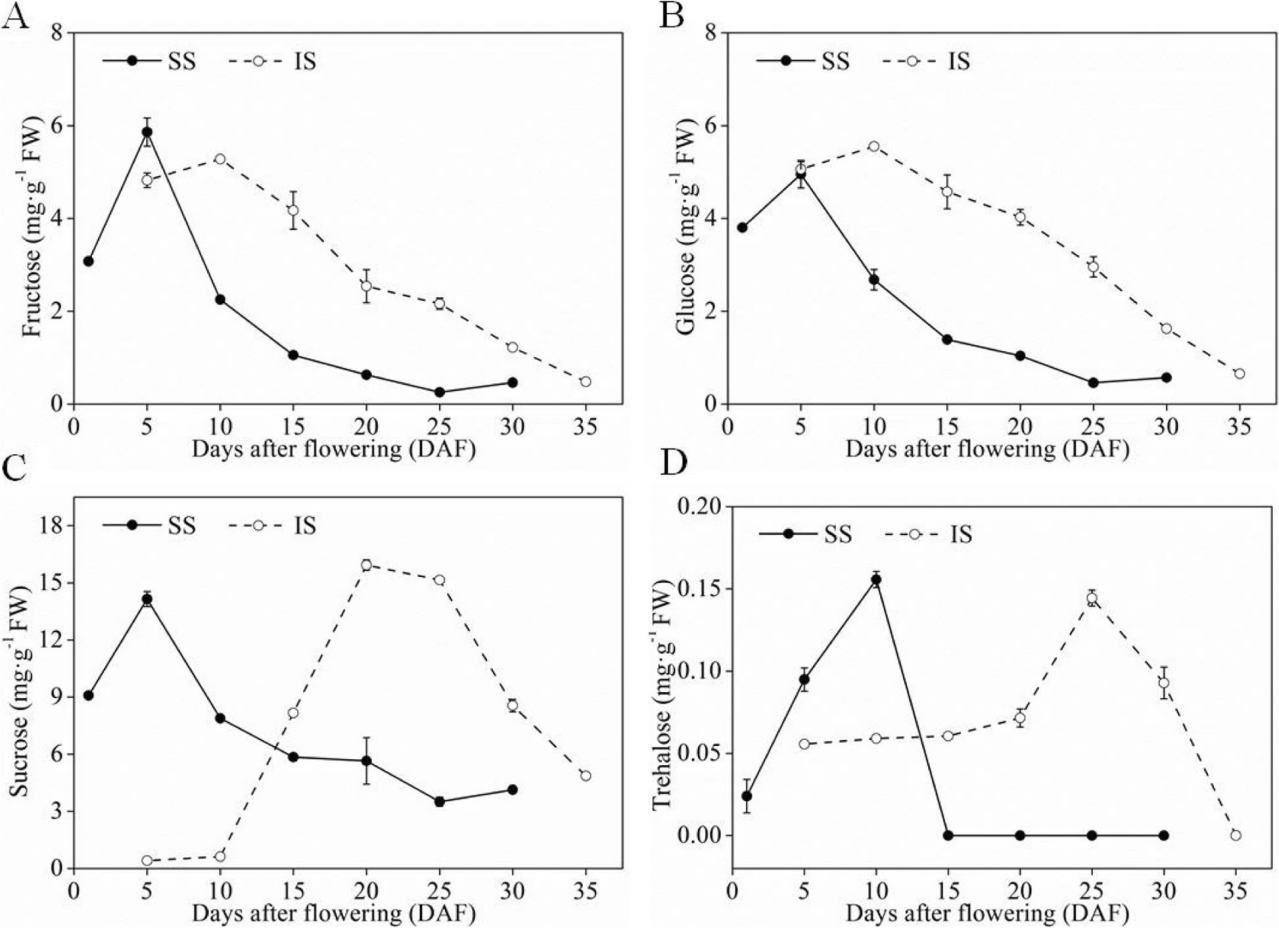
Contents of fructose (**A**), glucose (**B**), sucrose (**C**), and trehalose (**D**) of superior spikelets (SS) and inferior spikelets (IS). Data in the figure are means and S.E. of four independent measurements

GC-MS results showed that the soluble sugars were the main differential metabolites between SS and IS. In this study, HPLC was performed to further analyze the dynamic changes in the content of sucrose, fructose, glucose, and trehalose. As shown in Fig. 3, during the early grain-filling stage, an increase was observed in the content of all 4 soluble sugars in SS. The contents of sucrose, fructose, and glucose reached high levels at 5 DAF, while the content of trehalose reached a high level at 10 DAF. As the grain-filling process progressed, the levels of all 4 soluble sugars decreased. As for the rate of decrease, the contents of sucrose, fructose, and glucose in SS exhibited the largest decrease from 5 to 10 DAF, while the level of trehalose exhibited the largest decrease from 10 to 15 DAF, followed by a slow decrease. During the early grain-filling stage in IS, the contents of sucrose, glucose, and fructose also exhibited an increase. The contents of glucose and fructose peaked at 10 DAF and gradually decreased as the grain-filling process progressed, until the end of the grain-filling stage. From 10 to 20 DAF, the level of sucrose rapidly increased and peaked at 20 DAF. After this, the content of sucrose decreased, but at a slower rate compared to that in SS. The content of trehalose did not exhibit any changes during the early grain-filling stage and remained at a relatively low level. During the mid-grain-filling stage, which was 15–25 DAF, the content of trehalose began to increase and reached its peak at 25 DAF, followed by a gradual decrease.

**Fig. 4:**
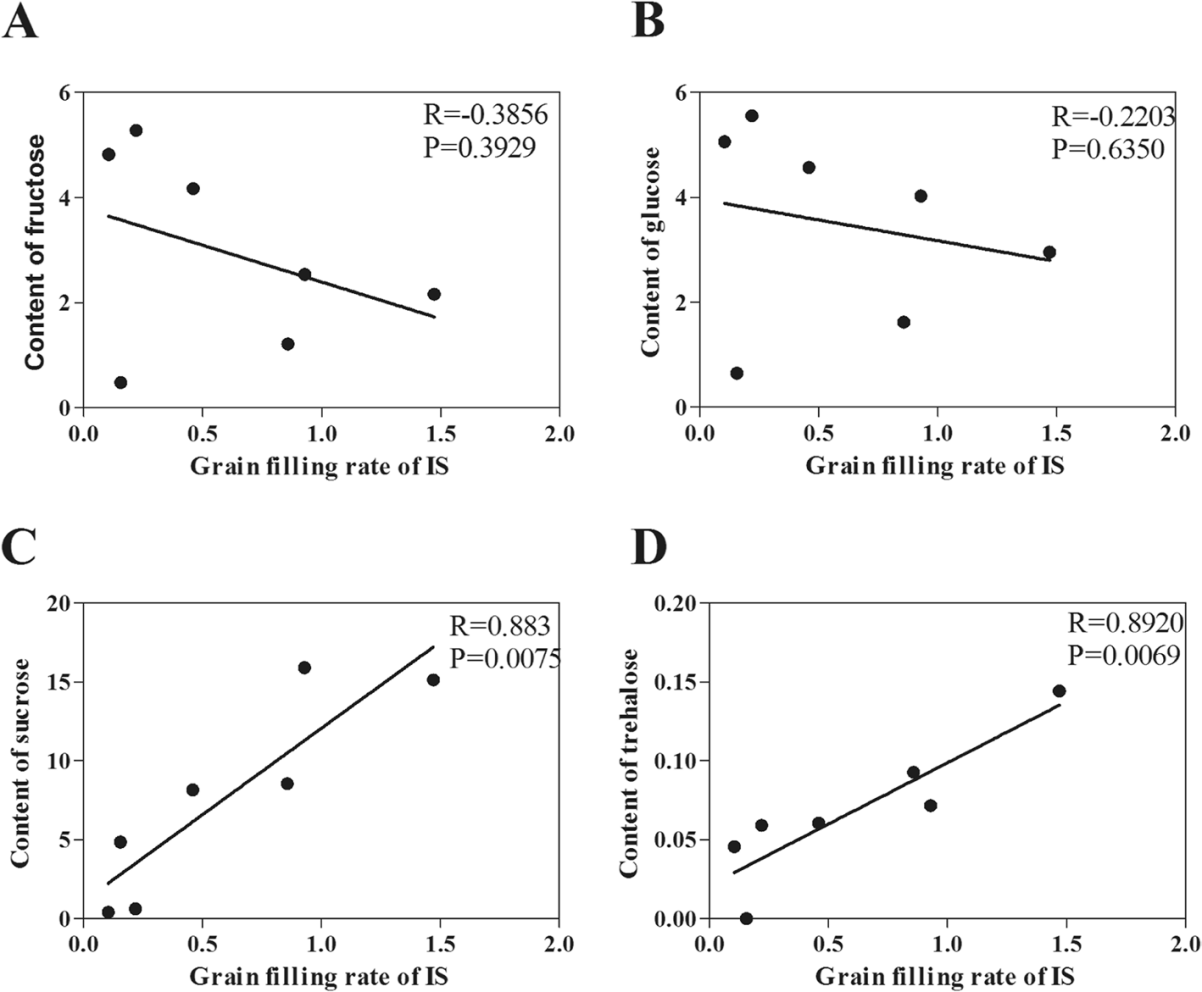
Correlation between contents of fructose, glucose, sucrose, and trehalose and the grain filling rate of inferior spikelets (IS). **A** Content of fructose was not associated with grain filling rate of IS (*R* = -0.3856, *P* = 0.3929). **B** Content of glucose was not associated with grain filling rate of IS (*R* = -0.2203, *P* = 0.6350). **C** Content of sucrose was positively associated with grain filling rate of IS (*R* = 0.883, *P* = 0.0075). **D** Content of trehalose was positively associated with grain filling rate of IS (*R* = 0.892, *P* = 0.0069). Pearson’s correlation was used to determine correlation coefficients (*R*) and *P*-values for each correlation

A comparison of the changes in the contents of the 4 soluble sugars between SS and IS revealed that during the grain-filling process, the contents of glucose and fructose had similar patterns in SS and IS. They increased in the early grain-filling stage, followed by a gradual decrease during the mid- and late grain-filling stages. The changes in the contents of sucrose and trehalose were consistent between SS and IS, exhibiting a peak in SS at the early grain-filling stage and in IS at the mid- and late grain-filling stages. Correlation analysis showed that both of the contents of sucrose and trehalose were positively associated with the grain filling rate of IS (Fig. 4). The results also showed that the correlation of trehalose with grain filling rate of IS (*r* = 0.892) was more than the correlation of sucrose with grain filling rate of IS (*r* = 0.883)(Fig. 4C, D).

### The effect of exogenous Trehalose and sucrose on grain filling

**Fig. 5:**
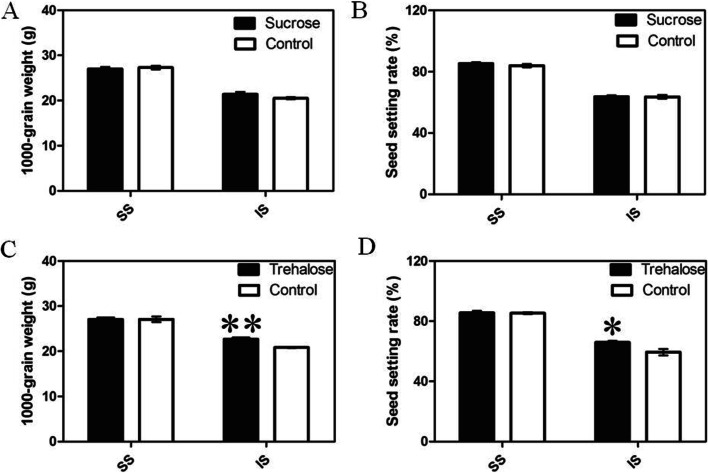
Effects of applied sucrose and trehalose on the 1000-grain weight and seed setting rate of superior spikelets (SS) and inferior spikelets (IS). Data in the figure are means and SD of four independent measurements. Asterisks represent significant difference (*P < 0.05, **P < 0.01)

**Fig. 6:**
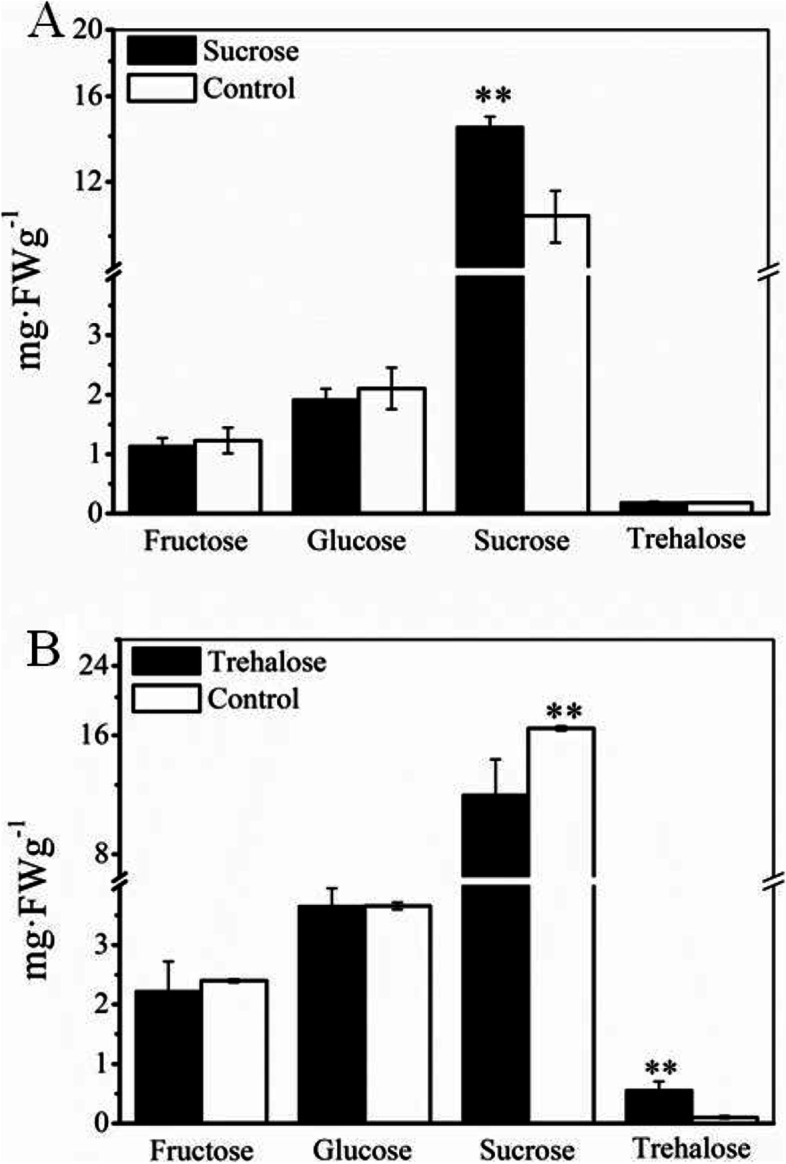
Effects of applied trehalose (**A**) and sucrose (**B**) on the contents of fructose, glucose, sucrose, and trehalose of inferior spikelets (IS). Data in the figure are means and SD of four independent measurements. Asterisks represent significant difference (**P < 0.01)

Exogenous trehalose and sucrose applications were performed to further investigate the functions of these two soluble sugars in the grain-filling process. Figure 5 showed that treatment with 100 mM trehalose could significantly increase the 1000-grain weight and seed setting rate in IS during the grain-filling period. When 100 mM sucrose was applied, the 1000-grain weight of IS increased but did not reach a significant level. Neither the sucrose nor the trehalose treatment had no effect on the grain filling of SS. Figure 6 showed that after the application of exogenous trehalose, the sucrose content in IS was significantly reduced while the contents of glucose and fructose were unaffected. Exogenous application of sucrose did not significantly alter the contents of the other three sugars.

### The regulation of starch synthesis in IS during grain filling by exogenous trehalose

**Fig. 7:**
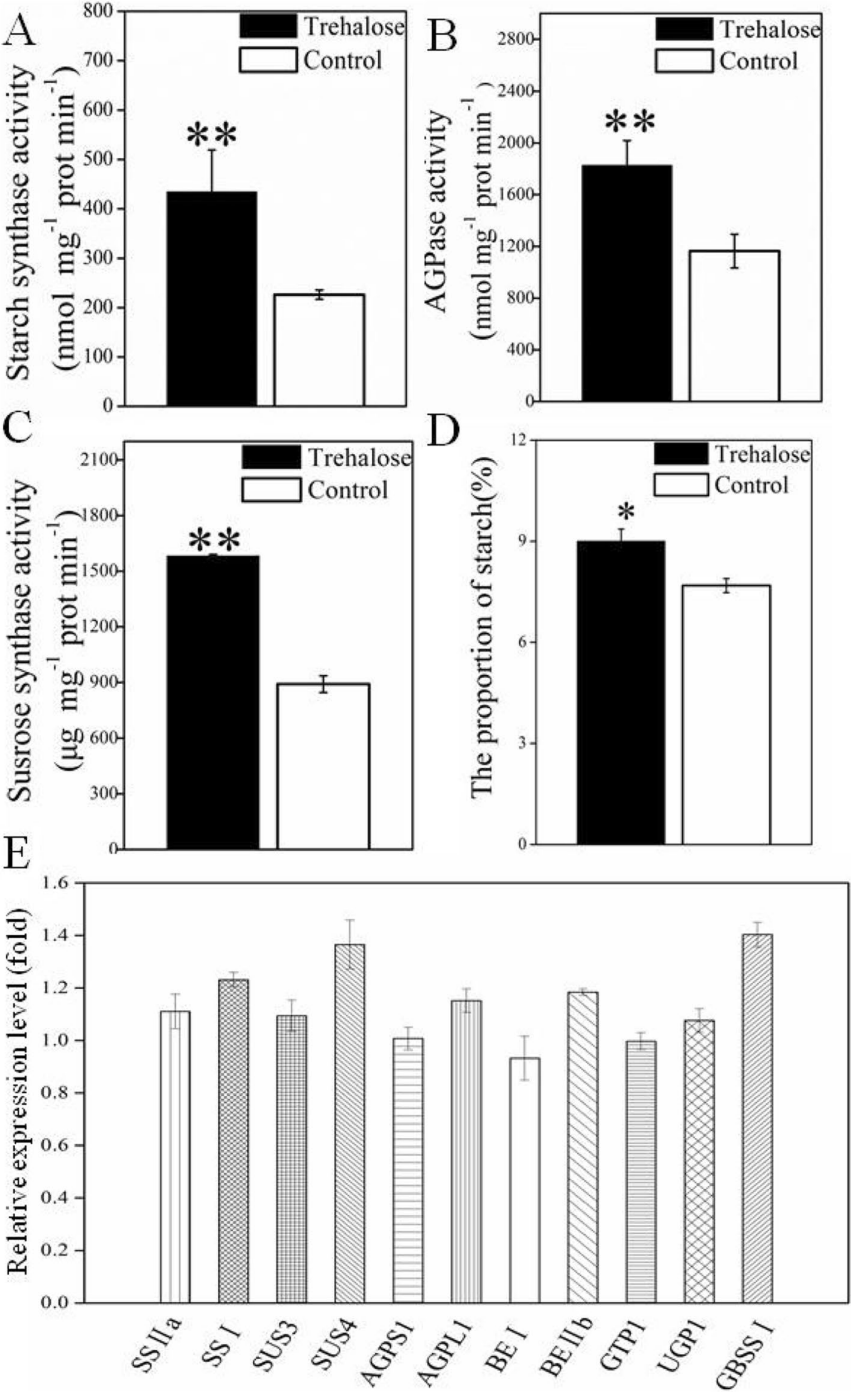
Effects of exogenous trehalose on starch synthesis of inferior spikelets (IS). **A** Starch content. **B** Starch synthase activity. **C** AGPase activity. **D** Susrose synthase activity. Data in the figure (**A**, **B**, **C**, **D**) are means and SD of four independent measurements. Asterisks represent significant difference (*P < 0.05, **P < 0.01). **E** Expression of starch synthesis-related genes in IS determined by qRT-PCR analysis. RNA was extracted from IS with trehalose treatment and control and then reverse transcribed for qRT-PCR analysis. The mRNA level of starch synthesis genes in IS with treatment were taken as 1-fold. The values are the means and SD of 6 replicates from two independent biological measurements. The PCR primers are shown in Table S4

Starch biosynthesis is considered to be the most important metablolic process during the rice grain filling. Figure 7A, B, C showed that after the application of exogenous trehalose, the activity of StSase, AGPase, and SuSase significantly increased in IS. At the same time, the content of starch in IS was also significantly elevated (Fig. 7D). A study conducted by Zhu et al. revealed that the differences in starch synthesis and metabolism between SS and IS were mainly caused by the differential expression of 11 genes involved in the conversion of sucrose and starch synthesis and metabolism during grain filling [9]. In this study, we utilized qRT-PCR to analyze the effect of exogenous trehalose on the expression of these 11 genes. Results showed that exogenous trehalose treatment increased the expression of starch synthase genes (*SS I, SS II a*), the sucrose synthase gene (*SUS4*), the starch branching enzyme gene (*BE II b*), the AGPase gene (*AGPL2*), and the *GBSSI* gene compared to the water treatment (Fig. 7E).

### Analysis of the differential spatiotemporal expression pattern of trehalose synthesis-related genes between SS and IS

**Fig. 8:**
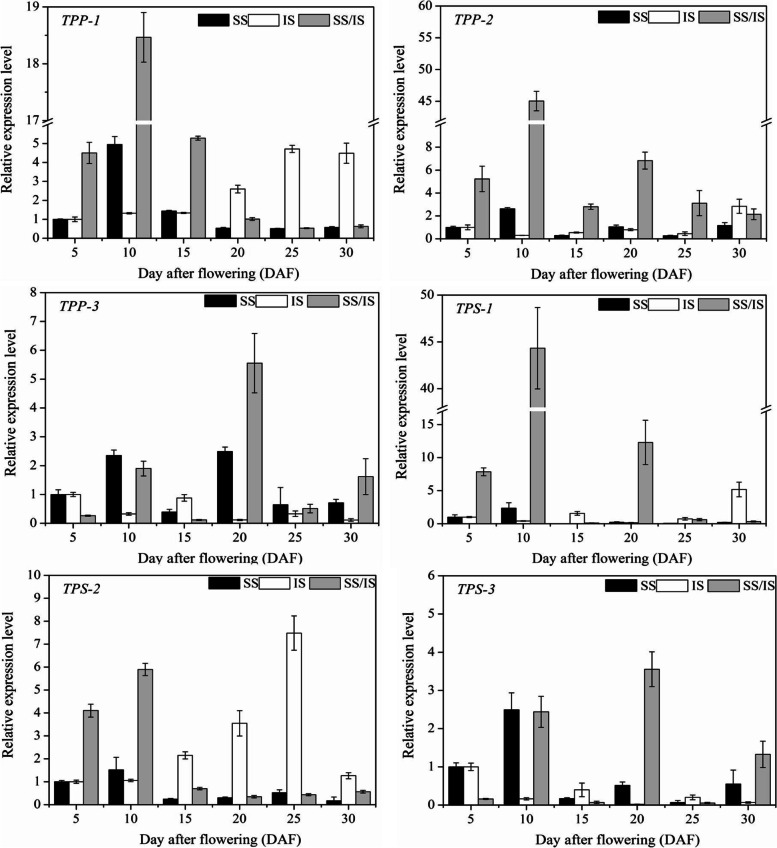
Differences in the expression of *trehalose-6-phosphate synthase (TPS)* and *trehalose-6-phosphate phosphatase (TPP)* gene family members between superior spikelets (SS) and inferior spikelets (IS). RNA was extracted from SS and IS at 5, 10, 15, 20, 25, and 30 DAF and then reverse transcribed for qRT-PCR analysis. The value of mRNA level in the 5 DAF SS and 5 DAF IS was set to 1×, and the other SS and IS were calculated relative to this value. The values are the means and SD of 6 replicates from two independent biological measurements. The PCR primers are shown in Table S5

A previous RNA-seq study identified 6 trehalose synthesis-related genes in rice spikelets, including the trehalose-6-phosphate synthase (TPS) gene family members *TPS-1*, *TPS-2*, and *TPS-3*, as well as trehalose-6-phosphate phosphatase (TPP) gene family members *TPP-1*, *TPP-2*, and *TP*P-3 [7]. Based on these results, we performed qRT-PCR to analyze the differential spatiotemporal expression patterns of the 6 genes mentioned above (Fig. 8). Our results showed that during grain filling in SS, the expressions of *TPS-1*, *TPS-2*, *TPS-3*, *TPP-1* and *TPP-2* reached their peaks at around 10 DAF and then gradually decreased during grain filling. During grain filling in the IS, *TPS-2* and *TPP-1* exhibited low expression levels at the early grain-filling stage; as grain filling progressed, the expression of *TPS-2* and *TPP-1* increased during the mid-grain-filling stage and decreased in the late grain-filling stage. During the early stage of grain filling, the expression of *TPS-1, TPS-2*, *TPP-1* and *TPP-2* was significantly higher in SS than in IS. In the late stages of grain filling, the expression levels of *TPS-1, TPS-2* and *TPP-1* were higher in IS than in SS.

## Discussion

### Soluble sugars are the main differential metabolites between SS and IS

The rice cultivar ‘Jinhui No. 809’ exhibited significant differences in grain filling between SS and IS. In the present study, the untargeted metabolomics analysis revealed a marked metabolites difference between the SS and IS. Except for a few metabolites including sucrose, trehalose, and hydroxyproline, the majority of the differential metabolites in IS had a higher level at 10 DAF than at 25 DAF, that is, as grain filling progressed, the levels of most soluble sugars and acid substances decreased, indicating that grain filling is a process converting soluble sugars, such as fructose and glucose, to polysaccharides, as well as converting amino acids to proteins. Between SS (active grain filling stage) and IS (developmental stagnancy phase) at 10 DAF, the contents of most soluble sugars and amino acids were higher in IS than in SS, indicating that the conversion of sugars and amino acids between SS and IS was unsynchronized. Interesting, a total of 35 differential metabolites have been identified between 10 DAF SS and 25 DAF IS, which may be related to the lower maximum grain-filling rate of IS compared with the SS.

A previous study showed that the rice grain-filling process is mainly a process of starch accumulation [15] and that the starch in the grains is mostly converted from sucrose [16]. The present results showed that the contents of soluble sugars were significantly higher than those of other metabolites. This indicated that soluble sugars were the major metabolites in the grains, and most sugars were related to the conversion of sucrose. As the foundation of substance and energy metabolism, sugars themselves and their intermediate metabolites are structural components for energy and storage substances, as well as the intermediates for synthesizing other organic molecules [17]. Sugars can also act as signaling molecules and can interact with inorganic regulatory networks to promote or inhibit plant growth [18–20]. The total contents of the four types of soluble sugars showed that at the early grain-filling stage, the contents of soluble sugar in SS was higher than in IS. However, this pattern was reversed at the late stage of grain filling (Fig. 3). During the early stage of fertilization, the contents of endogenous hormones such as ABA and IAA are at high levels in SS [21, 22]. During this time, the ability to allocate photosynthate was also high in SS; photoassimilates generated in the leaves were continuously transported into the grains. As a result, the contents of soluble sugars in SS were higher than in IS. As the grain-filling process progressed, the activity of enzymes involved in the conversion of sugar to starch gradually increased in SS, and the contents of sugar in the grains continued to decrease. However, at the early grain-filling stage, most genes involved in the conversion of sucrose to starch exhibited lower expression levels in IS than in SS [7, 8, 23]. The activity of enzymes involved in the conversion of sugar to starch was also lower in IS [24, 25], with a low efficiency in converting soluble sugars to starch. Thus, the level of soluble sugars in IS was lower than that in SS. At the mid- and late stages of grain filling, the expression level of genes related to the conversion of sucrose to starch, as well as the activity of enzymes involved in this process, gradually increased in IS [7, 9]. As a result, the level of soluble sugars continuously decreased. Since grain filling in SS was almost completed at this time, the level of soluble sugars in IS at the mid- and late stages of grain filling was always higher than that in SS at the same stage. However, the higher sugar content in IS was not favorable for the transport of photosynthate to IS, leading to a low seed setting rate in IS. These results indicated that the difference in the soluble sugar contents was an important cause of the difference in grain filling between SS and IS.

### The trehalose content was an important cause of the poor grain filling of IS

Correlation analyses indicated that the changes in sucrose and trehalose were positively corrected with the grain filling rate of IS (Fig. 4C, D), suggesting that the sucrose and trehalose may play important roles in regulating the grain filling of IS. Previous studies have shown that applying an appropriate content of exogenous sucrose induced the expression of sucrose synthase genes and increased the activity of sucrose synthase, thereby increasing the 1000-grain weight [26, 27]. In this study, 100 mM of exogenous sucrose solution was applied at 10 DAF. Results showed that the 1000-grain weight of IS increased, but not significantly (Fig. 5A). This is possibly because the exogenous sucrose content did not reach a content high enough to promote grain filling of rice cultivar ‘Jinhui No. 809’. However, when applying the same content of trehalose solution, the 1000-grain weight and seed setting rate of IS were significantly increased (Fig. 5C, D).

Trehalose and sucrose are both disaccharides; their metabolism is also similar. Trehalose, a non-reducing sugar composed of two glucose molecules, is present in many plants and participates in signal regulatory processes [28, 29]. In this study, we found that the timing when the trehalose content reached its peak in SS and IS was later than that of sucrose. The sucrose content in IS rapidly increased after 10 DAF, but the trehalose content remained at a low level. At this time, grain filling in IS had not been activated. At 15 DAF, the level of trehalose in IS began to slowly increase, followed by the activation of grain filling in IS. Starch contents gradually increased in the grains (Fig. 1D), and the trehalose content in IS reached its maximum at 25 DAF (Fig. 3D), consistent with the timing of the grain-filling rate reaching its maximum (25 DAF) in IS (Fig. 1C). In addition, the results from this study showed that the exogenous application of sucrose did not affect the trehalose content in IS. However, after the application of exogenous trehalose, the content of sucrose significantly decreased and the starch content significantly increased in the grains (Fig. 6). This indicated that the trehalose may play an important role in regulating grain filling of IS and can promote the conversion of sucrose to starch.

Results from qRT-PCR showed that trehalose induced the expression of key genes involved in sucrose conversion and starch synthesis in IS. The enzyme activities of SuSase, StSase, and AGPase also significantly increased (Fig. 7). In *Arabidopsis*, exogenous trehalose has been found to induce the expression of *APL3*, a gene involved in starch synthesis, resulting in starch accumulation in the cotyledon [30]. These results showed that certain content of trehalose in the grain-filling stage can activate the expression of genes related to starch synthesis and promote the conversion of sucrose to starch. The asynchronous change in trehalose content between SS and IS is likely an important reason leading to the differential spatial and temporal patterns of expression of genes related to starch synthesis.

### *TPS-2* and *TPP-1* are key genes that cause the asynchronous change in the trehalose content between SS and IS

Trehalose synthesis in plants is a two-step process: the production of trehalose-6-phosphate (T6P) from UDP-Glucose and Glucose-6-phosphate, catalyzed by TPS, and consecutive dephosphorylation of T6P to trehalose, catalyzed by TPP [28]. In this study, we found that the changes in *TPS-2* and *TPP-1* gene expression during grain filling in IS were similar to the pattern of changes in the trehalose content, that is, the expression levels of *TPS-2* and *TPP-1* were low at the at the early grain-filling stage and increased from the mid-grain-filling stage. Furthermore, the expression patterns of *TPS-2* and *TPP-1* also showed a similar pattern as the changes in the trehalose content between SS and IS, which were higher in SS at early grain filling stage and lower in late grain-filling stage (Fig. 8). Therefore, the differential expression of *TPS-2* and *TPP-1* between SS and IS is predicted to be an important reason leading to the asynchronous change in the trehalose content between SS and IS. In addition, a TPS isoform was identified as a target protein of the 14-3-3 protein [31]. 14-3-3 proteins bind to phosphorylated motifs and function in multiple developmental processes by regulating the activity of a wide variety of target proteins [32]. In vivo phosphorylation of Ser22 and Thr49 of the TPS protein by AMPK and SnRK1s resulted in the binding of TPS to the 14-3-3 protein [33]. Interesting, our previous study determined that the 14-3-3 protein showed temporal and spatial differences in expression patterns between SS and IS; specifically, reducing the expression of 14-3-3 protein during grain filling effectively promoted starch synthesis in IS [34]. Based on these results, it was speculated that through the binding of phosphorylated TPS-2 or TPP1 protein, the 14-3-3 protein could regulate trehalose synthesis in SS and IS. However, the mechanism needs to be further investigated.

## Conclusions

In summary, soluble sugars were a major type of differential metabolite between SS and IS. Among them, trehalose was an important sugar signal that regulated the difference in starch synthesis between SS and IS, while *TPS-2* and *TPP-1* were another important reason leading to the asynchronous change in trehalose content between SS and IS. Future studies should focus on the molecular mechanism that causes the differential expression of *TPS-2* and *TPP-1* between SS and IS.

## Methods

### Plant material and sampling

In this study, plant material of *Oryza sativa* L. subsp. Indica variety ‘Jinhui No. 809’ was acquired from Fujian Agriculture and Forestry University (FAFU), China (119.280 E, 26.080 N). The experiments were carried out in the experimental field at the FAFU during the rice-growing season in 2018-2019. The germinated seeds were planted in a paddy field. The 5-leaf stage seedlings were transplanted into the filed, and the spacing used for rice transplantation was 0.15 × 0.15 m, with one seedling per hill. The panicles that headed on the same day were tagged. The flowering date and position of each spikelet on the tagged panicles were recorded. The tagged panicles were sampled every 5 days from 1 to 35 DAF. According the previous report [2], spikelets on the first and second primary rachis branches counted from the top of the panicle were collected as SS, and spikelets on the base of secondary rachis branches of the lowest primary branch were collected as IS. The samples of SS and IS were frozen in liquid nitrogen immediately after collection and were stored at − 80 °C.

### Metabolite extraction and GC-MS analysis

Extraction of total metabolites was performed using a modified version of the method used by previous report [35]. 200 mg of dehulled grains was extracted at 70 °C temperature with a mixture of solvents (pre-cooled; 1.4 ml of methanol, 60 μl of 0.2 mg ml^− 1^ ribitol) for 30 min. The sample was then centrifuged at 13,000 rpm for 10 min at room temperature. After the supernatant was collected, 750 μl of pre-cooled chloroform was added and the mixture was shaken for 5 min. For phase separation, 1.4 ml of pre-cooled H_2_O was added to each sample, vortexed, and centrifuged (15 min, 4000 rpm). From the upper polar phase, ≈1 ml was collected, dried completely, and derivatized for the GC-MS analysis, and the remaining extract was used for sucrose, fructose, glucose, and trehalose determination using high performance liquid chromatography (HPLC).

Metabolites sample used for GC-MS analysis were derivatized by methoxyamination and trimethylsilylation. Dried polar phases were shaken for 2 h at 37 °C in 50 μl of MeOX (20 mg ml^− 1^ methoxyaminhydrochloride in pyridine) followed by 1 h of shaking at 37 °C in 80 μl of methyl-trimethylsilyl trifluoroacetamide (MSTFA) mixture. Metabolite profiling of rice spikelets analysis by GC-MS was performed as exactly described in Kusano et al. [36]. For each sample, 10 biological replicates were prepared for metabolite profiling. An equivalent of 6 μg of the derivatized samples was injected into the GC-MS instrument (TQ8040, Shimadzu, Japan). A retention time (RT) correction was performed for all the samples. The RT was then used as a reference against which the remaining spectra were queried and a file containing the abundance information for each metabolite in all the samples was assembled. The metabolites from the GC-MS spectra were identified by searching in National Institute of Standards and Technology (NIST) library using NIST MS Search 2.0. The data were normalized to the intensities of added internal standards, and then exported to SIMCA 13.0 software (Umetrics, Umea, Sweden) for multivariate statistical analysis. After principal component analysis (PCA), Orthogonal partial least squares discriminant analysis (OPLS) models were used to visualize the high-dimensional data and determine the metabolites differences between SS and IS. The candidate metabolites were identified according to statistically significant threshold of variable influence on projection (VIP) values obtained from the OPLS model (VIP > 1.0) and a *p*-value < 0.05 (obtained from two-tailed Student’s t-test).

### Determination of sucrose, glucose, fructose and trehalose

Soluble sugars, specifically sucrose, glucose, fructose, and trehalose, were measured using HPLC. The soluble sugars present in the samples were identified and quantified by comparing each peak retention times and areas of the HPLC profiles with those of known standards. The amount of each soluble sugar was calculated by using recovery ratio of the internal standard. Stock solutions of glucose, fructose, sucrose, and trehalose at contents of 100, 100, 500, and 10 mg/mL, respectively, were prepared or the HPLC system. A standard curve for each sugar was constructed from different dilutions of the stock solution. Glucose, fructose, sucrose, and trehalose were obtained from Solarbio Life Science (Beijing, China). The extract was injected into the HPLC (Shimadzu, Japan), which was equipped with a pulseless pump (LC-20 AD, Shimadzu, Japan) with a 1.0 ml min^− 1^ flow rate and an refractive index detector (RID-10A, Shimadzu, Japan). A 75/25 acetonitrile/water mixture was used as the mobile phase. A Shodex column (Asahipak NH2P-50 4E, 250 mm × 4.6 mm) was used for the separation of sucrose, glucose, fructose, and trehalose, and the analyses were conducted with a chromatogram.

### Exogenous sucrose and trehalose applications

Based on the Tang et al. study [26], the 100 mM trehalose and sucrose were sprayed on the tagged panicles at 10 DAF. The chemical was applied daily for 5 d at the rate of 1 ml panicle^− 1^ for each application. The solution contained Tween 20 at a final content of 0.01% (v/v). Panicles sprayed with the same volume of deionized water containing the same contents of Tween 20 were used as the control. The chemical treatment contained 40 tagged panicles with three replications. At 15 DAF, 15 tagged panicles for every replication were collected and used for the determination of soluble sugar, starch, and enzyme activity. The remaining panicles were used for the determination of the seed setting rate and the 1000-grain weight at maturity.

### Enzyme activity assays

The enzyme activities of sucrose synthase (SuSase), ADP-glucose pyrophosphorylase (AGPase) and starch synthase (StSase) were measured using enzyme assay kits (Cominbio, Suzhou, China). Enzyme activities of all the samples were expressed as μg·mg^− 1^prot·min^− 1^ for SuSase and nmol·mg^− 1^prot·min^−1^ for AGPase and StSase.

### Real-time reverse transcription PCR (qRT-PCR)

Total RNA extraction from dehulled grains using the TRIzol reagent (Invitrogen, Shanghai, China). 1 μg of DNA-free RNA was reverse transcribed to cDNA using MMLV reverse transcriptase (TaKaRa, Kyoto, Japan). The Beacon Designer 7.0 software (Palo Alto, CA, USA) was used to design the specific primer pairs. All the primers listed in the Table S4 and S5. Gene expression was assessed using a Mastercycler EP Realplex RT-PCR system (Eppendorf, Hamburg, Germany) and Bestar™ Real-time PCR Master Mix SYBR Green (DBI Bioscience, Shanghai, China). To determine relative fold differences of each genes in each samples, the Ct value for the genes studied was normalized to the Ct value for *18SrRNA*, and the relative expression was calculated using the 2^−ΔΔCt^ method.

## Supplementary Information

**Additional file 1: Figure S1.** Principal component analysis (PCA), Orthogonal projections to latent structures (OPLS) and S-plot analysis of the superior spikelets (SS) and inferior spikelets (IS) metabolomes. (A) PCA model of 10 DAF IS and SS. (B) PCA model of 10 DAF IS and 25 DAF IS. (C) PCA model of 25 DAF IS and 10 DAF SS. (D) OPLS model of 10 DAF IS and SS. (E) OPLS model of 10 DAF IS and 25 DAF IS. (F) OPLS model of 25 DAF IS and 10 DAF SS. (G) S-plot of 10 DAF IS and SS. (H) S-plot of 10 DAF IS and 25 DAF IS. (I) S-plot of 25 DAF IS and 10 DAF SS.

**Additional file 2: Table S1.** Significantly differential metabolites between 10 DAF of superior spikelets (SS) and inferior spikelets (IS).

**Additional file 3: Table S2.** Significantly differential metabolites between 10 DAF and 25 DAF of inferior spikelets (IS).

**Additional file 4: Table S3.** Significantly differential metabolites between 10 DAF of superior spikelets (SS) and 25 DAF of inferior spikelets (IS).

**Additional file 5: Table S4.** Primer sequences of genes related to sugar conversion and starch synthesis.

**Additional file 6: Table S5.** Primer sequences of genes related to trehalose synthesis.

## Data Availability

The datasets generated and analyzed in the current study are available from the corresponding author on reasonable request. All data generated or analyzed during this study are included in this published article and its Supplementary information files.

## Abbreviations

SS: Superior spikelets
IS: Inferior spikelets
GC-MS: Gas chromatography-mass spectrometry
MSTFA: Methyl-trimethylsilyl trifluoroacetamide
HPLC: High performance liquid chromatography
RT: Retention time
NIST: National Institute of Standards and Technology
OPLS-DA: Orthogonal partial least squares discriminant analysis
VIP: Variable influence on projection
StSase: Starch synthase
AGPase: ADP-glucose pyrophosphorylase
SuSase: Sucrose synthase
qRT-PCR: Real-time reverse transcription PCR
DAF: Days after flowering
TPS: Trehalose-6-phosphate synthase
TPP: Trehalose-6-phosphate phosphatase
T6P: Trehalose-6-phosphate
