# Supplementary figures and images for: GC-MS-based metabolite profiling of key differential metabolites between superior and inferior spikelets of rice during the grain filling stage
Source: BMC Plant Biol. 2021 Sep 28;21:439. doi: 10.1186/s12870-021-03219-8 (PMC8477532; doi:10.1186/s12870-021-03219-8)

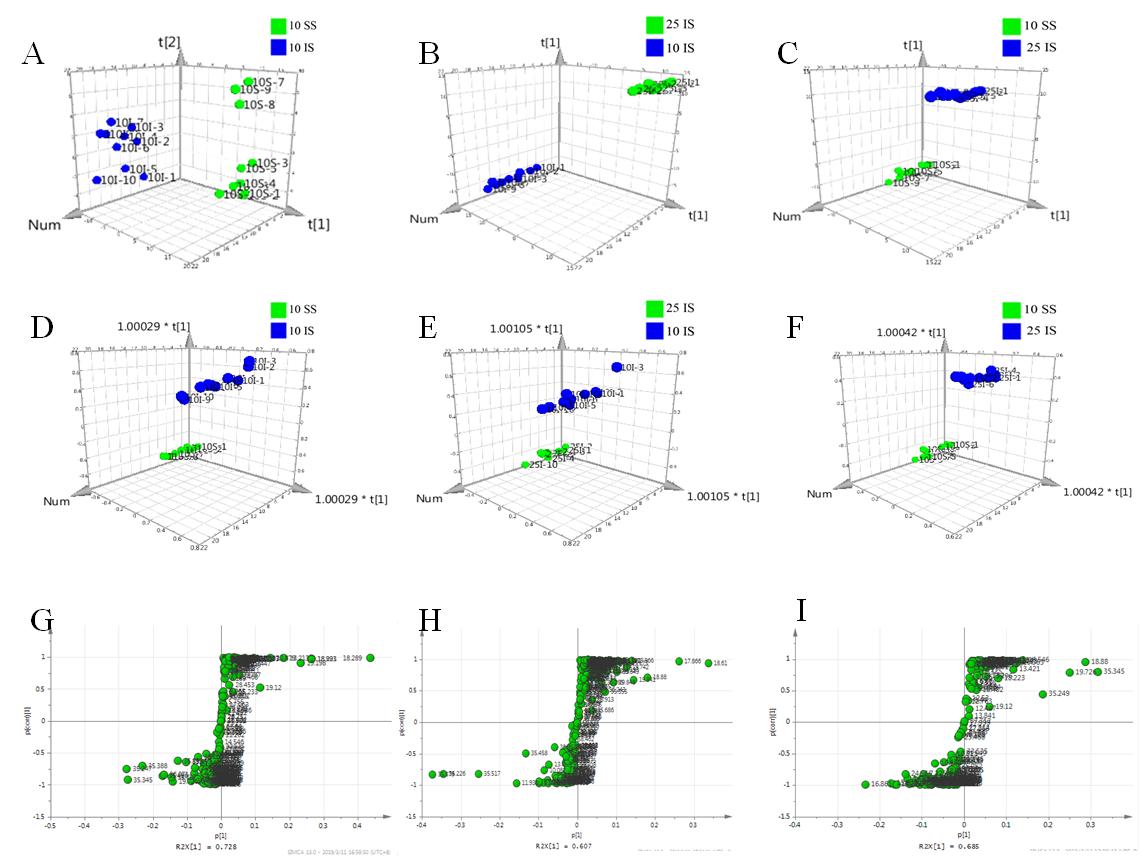
Fig s1

Supplement: Supplementary file 1 — Additional file 1: Figure S1. Principal component analysis (PCA), Orthogonal projections to latent structures (OPLS) and S-plot analysis of the superior spikelets (SS) and inferior spikelets (IS) metabolomes. (A) PCA model of 10 DAF IS and SS. (B) PCA model of 10 DAF IS and 25 DAF IS. (C) PCA model of 25 DAF IS and 10 DAF SS. (D) OPLS model of 10 DAF IS and SS. (E) OPLS model of 10 DAF IS and 25 DAF IS. (F) OPLS model of 25 DAF IS and 10 DAF SS. (G) S-plot of 10 DAF IS and SS. (H) S-plot of 10 DAF IS and 25 DAF IS. (I) S-plot of 25 DAF IS and 10 DAF SS. [file 12870_2021_3219_MOESM1_ESM.doc]
