# Supplementary material for: GC-MS-based metabolite profiling of key differential metabolites between superior and inferior spikelets of rice during the grain filling stage
Source: BMC Plant Biol. 2021 Sep 28;21:439. doi: 10.1186/s12870-021-03219-8 (PMC8477532; doi:10.1186/s12870-021-03219-8)
Supplement: Supplementary file 5 — Additional file 5: Table S4. Primer sequences of genes related to sugar conversion and starch synthesis. [file 12870_2021_3219_MOESM5_ESM.doc]

Table S4 Primer sequences of genes related sugar conversion and starch synthesis

| Gene | Primers | Sequences (5’–3’) |
| --- | --- | --- |
| *AGPS1*  (LOC_Os09g12660.1) | AGPS1-UP | ATGAGGGTGCAAGCTTACTTAT |
|  | AGPS1-Down | CTTTTGATGGAGGCAAGTATCG |
| *AGPL2*  (LOC_Os09g12660.1) | AGPL2-UP | GGAAAGATTGAATATTGGGGGC |
|  | AGPL2-Down | TCAGAGGAAAGAGTTGAACTCC |
| *SSⅠ*  (LOC_Os06g06560.1) | SSⅠ-UP | CTCCCGGAAGAGTGTATTGAAT |
|  | SSⅠ-Down | CCTGCAATTCAGCTTTACACTT |
| *SSⅡa*  (LOC_Os06g12450.1) | SSⅡa-UP | GAATTTGCAGAGGATAAGAGCG |
|  | SSⅡa-Down | CGATCACGTTCATGACATTCTC |
| *GBSSⅠ*  (LOC_Os06g04200.3) | GBSSⅠ-UP | GTGAGGTTTTTCCATTGCTACA |
|  | GBSSⅠ-Down | GTAGATCTTCTCACCGGTCTTT |
| *BEⅠ*  (LOC_Os06g51084.1) | BEⅠ-UP | ATGGTCTAAATGGCTATGACGT |
|  | BEⅠ-Down | AATTGGCATAGTTGAACAGACG |
| *BEⅡb*  (LOC_Os02g32660.1) | BEⅡb-UP | GATCAGTATGAAGGAGGACTGG |
|  | BEⅡb-Down | ACCTACTAATGCTGCAGAATGT |
| *SuS3*  (LOC_Os07g42490.1) | SuS3-UP | CAGATCATTGCTGAGTACAACG |
|  | SuS3-Down | TTGATCCTCAGATACTCCCAGA |
| *SuS4*  (LOC_Os03g22120.1) | SuS4-UP | TCGAGCAGCTCACCGTATC |
|  | SuS4-Down | CAAGTATGTACGGATCGTTGTAC |
| *UGP1*  (LOC_Os09g38030.1) | UGP1-UP | GTACCCTCATCTCTTACGAAGG |
|  | UGP1-Down | AGTTTCGAGTTGCAGAACTTTC |
| *GPT1*  (LOC_Os08g08840.2) | GPT1-UP | TCTGTCAGTGGCATGAATTACT |
|  | GPT1-Down | TAAGTGGTAGAAAACGCTCTGT |
