# Supplementary material for: GC-MS-based metabolite profiling of key differential metabolites between superior and inferior spikelets of rice during the grain filling stage
Source: BMC Plant Biol. 2021 Sep 28;21:439. doi: 10.1186/s12870-021-03219-8 (PMC8477532; doi:10.1186/s12870-021-03219-8)
Supplement: Supplementary file 6 — Additional file 6: Table S5. Primer sequences of genes related to trehalose synthesis. [file 12870_2021_3219_MOESM6_ESM.doc]

Table S5 Primer sequences of gene related trehalose synthesis

| Gene | Primers | Sequences (5’–3’) |
| --- | --- | --- |
| *TPS-2*  (LOC_Os08g34580.1) | TPS-2-UP | CAGGATACCGTACTTCTACACC |
|  | TPS-2-Down | GACAAACTCTGACACGATGATG |
| *TPS-1*  (LOC_Os09g20990.1) | TPS-1-UP | CATAGAGACAAAGGAGAGTGGG |
|  | TPS-1-Down | CGATCTGATGACCACGTTTAAC |
| *TPS-3*  (LOC_Os05g44210.2) | TPS-3-UP | GGAAATCGTGCACCAATACTAC |
|  | TPS-3-Down | CGTTGAACTGTGACTCGAAATT |
| *TPP-1*  (LOC_Os01g53000.1) | TPP-1-UP | CTCACATGATGTTGCCTATTGG |
|  | TPP-1-Down | CGGTCAAGAGCAACTACTCTAA |
| *TPP-2*  (LOC_Os05g44100.1) | TPP-2-UP | CGCAGATAGAAATAACACCGTG |
|  | TPP-2-Down | CACCAGTGCAGTTTCTTTAGTC |
| *TPP-3*  (LOC_Os09g23350.1) | TPP-3-UP | GAGCACACATGATGTTGGATAC |
|  | TPP-3-Down | AAACCTTAGCCCGAATCCTATC |
